# Spectacles and Eyeglasses

**Published:** 1903-01

**Authors:** 


SPECTACLES AND EYEGLASSES. Their Forms, Mounting, and Proper Adjustment. By R. J. PHILLIPS, M D , Ophthalmologist Presbyterian Orphanage, Philadelphia. Third edition revised, with 52 illustrations Philadelphia: P. Blakiston's Son & Co. 1902. [Price, $1.00 ]

This useful little book of 109 pages should be in the hands of every ophthalmologist, whether he furnishes glasses or writes prescriptions for them. The third edition is a credit to the author being quite complete and giving useful details without unnecessary padding to add to the size of the volume. The text remains very much the same with an addition wherever necessary to report the advances made in lens' manufacture and one or two new tables for reference. R.H.S.

